# 5-(4-Meth­ylphenyl)-1,3,4-thia­diazol-2-amine

**DOI:** 10.1107/S1600536809012434

**Published:** 2009-04-08

**Authors:** Jian-Ning Guan, Rong Wan, Yao Wang, Feng Han, Cheng-Zhen Xu

**Affiliations:** a Department of Applied Chemistry, College of Science, Nanjing University of Technology, No.5 Xinmofan Road, Nanjing, Nanjing 210009, People’s Republic of China

## Abstract

The title compound, C_9_H_9_N_3_S, was synthesized by the reaction of 4-methyl-benzoic acid and thio­semicarbazide. The thia­diazol ring adopts a planar conformation and makes a dihedral angle of 31.19 (18)° with the phenyl ring. In the crystal, mol­ecules are linked by N—H⋯N hydrogen bonds.

## Related literature

For applications of thia­diazole ligands, see: Nakagawa *et al.* (1996 ▶); Wang *et al.* (1999 ▶); Han *et al.* (2007 ▶). For bond-length data, see: Allen *et al.* (1987 ▶).
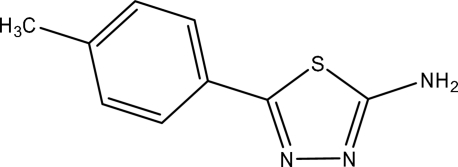


## Experimental

### 

#### Crystal data

C_9_H_9_N_3_S
*M* *_r_* = 191.25
Monoclinic, 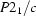

*a* = 12.284 (3) Å
*b* = 7.3730 (15) Å
*c* = 11.263 (2) Å
β = 109.09 (3)°
*V* = 964.0 (3) Å^3^
*Z* = 4
Mo *K*α radiation
μ = 0.29 mm^−1^
*T* = 293 K
0.30 × 0.10 × 0.10 mm

#### Data collection

Nonius CAD4 diffractometer
Absorption correction: ψ scan (North *et al.*, 1968 ▶) *T*_min_ = 0.918, *T*_max_ = 0.972
1963 measured reflections
1875 independent reflections
1351 reflections with *I* > 2σ(*I*)
*R*_int_ = 0.067
3 standard reflections every 200 reflections intensity decay: 1%

#### Refinement

*R*[*F* ^2^ > 2σ(*F* ^2^)] = 0.059
*wR*(*F* ^2^) = 0.181
*S* = 1.01
1875 reflections
118 parameters
H-atom parameters constrained
Δρ_max_ = 0.26 e Å^−3^
Δρ_min_ = −0.43 e Å^−3^

### 

Data collection: *CAD-4 EXPRESS* (Enraf–Nonius, 1989 ▶); cell refinement: *CAD-4 EXPRESS*; data reduction: *XCAD4* (Harms & Wocadlo,1995 ▶); program(s) used to solve structure: *SHELXS97* (Sheldrick, 2008 ▶); program(s) used to refine structure: *SHELXL97* (Sheldrick, 2008 ▶); molecular graphics: *SHELXTL* (Sheldrick, 2008 ▶); software used to prepare material for publication: *SHELXL97*.

## Supplementary Material

Crystal structure: contains datablocks global, I. DOI: 10.1107/S1600536809012434/at2758sup1.cif

Structure factors: contains datablocks I. DOI: 10.1107/S1600536809012434/at2758Isup2.hkl

Additional supplementary materials:  crystallographic information; 3D view; checkCIF report

## Figures and Tables

**Table 1:** Hydrogen-bond geometry (Å, °)

*D*—H⋯*A*	*D*—H	H⋯*A*	*D*⋯*A*	*D*—H⋯*A*
N3—H3*A*⋯N2^i^	0.86	2.13	2.970 (5)	166
N3—H3*B*⋯N1^ii^	0.86	2.18	3.025 (4)	166

Symmetry codes: (i) 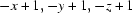; (ii) 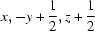.

**Fig. 1.**
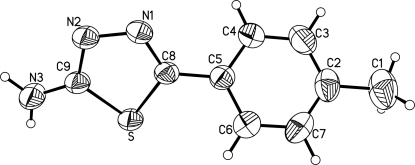
A view of the molecular structure of (I). Displacement ellipsoids are drawn at the 50% probability level.

**Fig. 2.**
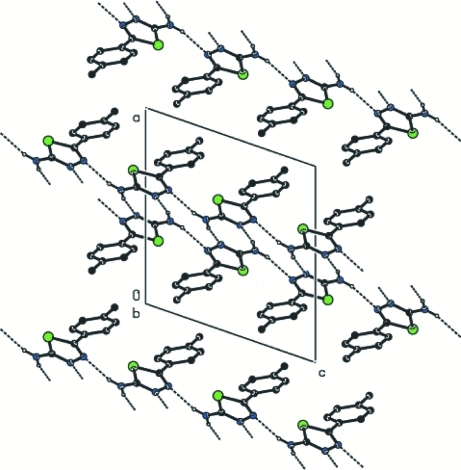
View of the N—H···N hydrogen bonds (dashed lines) in the unit cell. Dashed lines indicate hydrogen bonds.

## supplementary crystallographic information

### Comment

1,3,4-Thiadiazole derivatives represent an interesting class of compounds possessing broad spectrum biological activities (Nakagawa *et al.*, 1996; Wang *et al.*, 1999). These compounds are known to exhibit diverse biological effects, such as insecticidal, fungicidal activities (Wang *et al.*, 1999). We are focusing our synthetic and structural studies on thiadiazole derivatives and we have recently published the structure of 5-*m*-tolyl-[1,3,4]thiadiazol-2-ylamine (Han *et al.*, 2007). We report here the crystal structure of the title compound, (I).

In (I) (Fig. 1), the bond lengths and angles are within normal ranges (Allen *et al.*, 1987). The thiadiazole and the phenyl ring make a dihedral angle of 31.19 (18)°. The molecules link by N—H···N hydrogen bonds to stabilize the crystal structure (Fig. 2).

### Experimental

4-Methyl-benzoic acid (5 mmol) and thiosemicarbazide (5 mmol) were added in toluene (50 ml), which is heated under reflux for 4 h. The reaction mixture was left to cool to room temperature, poured into ice water, filtered, and the filter cake was crystallized from acetone to give pure compound (I). Crystals of (I) suitable for X-ray diffraction were obtained by slow evaporation of an acetone solution.

### Refinement

All H atoms were placed geometrically with C—H = 0.93–0.96 Å and N—H = 0.86 Å, and included in the refinement in riding motion approximation with *U*_iso_(H) = 1.2 or 1.5*U*_eq_ of the carrier atom.

### Crystal data

**Table:**

C_9_H_9_N_3_S	*F*(000) = 400
*M**_r_* = 191.25	*D*_x_ = 1.318 Mg m^−^^3^
Monoclinic, *P*2_1_/*c*	Melting point = 476–478 K
Hall symbol: -P 2ybc	Mo *K*α radiation, λ = 0.71073 Å
*a* = 12.284 (3) Å	Cell parameters from 25 reflections
*b* = 7.3730 (15) Å	θ = 9–13°
*c* = 11.263 (2) Å	µ = 0.29 mm^−^^1^
β = 109.09 (3)°	*T* = 293 K
*V* = 964.0 (3) Å^3^	Block, colourless
*Z* = 4	0.30 × 0.10 × 0.10 mm

### Data collection

**Table:**

Nonius CAD4 diffractometer	1351 reflections with *I* > 2σ(*I*)
Radiation source: fine-focus sealed tube	*R*_int_ = 0.067
graphite	θ_max_ = 26.0°, θ_min_ = 1.8°
ω/2θ scans	*h* = −15→0
Absorption correction: ψ scan (North *et al.*, 1968)	*k* = 0→9
*T*_min_ = 0.918, *T*_max_ = 0.972	*l* = −13→13
1963 measured reflections	3 standard reflections every 200 reflections
1875 independent reflections	intensity decay: 1%

### Refinement

**Table:**

Refinement on *F*^2^	Primary atom site location: structure-invariant direct methods
Least-squares matrix: full	Secondary atom site location: difference Fourier map
*R*[*F*^2^ > 2σ(*F*^2^)] = 0.059	Hydrogen site location: inferred from neighbouring sites
*wR*(*F*^2^) = 0.181	H-atom parameters constrained
*S* = 1.00	*w* = 1/[σ^2^(*F*_o_^2^) + (0.1*P*)^2^ + 0.5*P*] where *P* = (*F*_o_^2^ + 2*F*_c_^2^)/3
1875 reflections	(Δ/σ)_max_ < 0.001
118 parameters	Δρ_max_ = 0.26 e Å^−^^3^
0 restraints	Δρ_min_ = −0.42 e Å^−^^3^

### Special details

**Table:**

Geometry. All e.s.d.'s (except the e.s.d. in the dihedral angle between two l.s. planes) are estimated using the full covariance matrix. The cell e.s.d.'s are taken into account individually in the estimation of e.s.d.'s in distances, angles and torsion angles; correlations between e.s.d.'s in cell parameters are only used when they are defined by crystal symmetry. An approximate (isotropic) treatment of cell e.s.d.'s is used for estimating e.s.d.'s involving l.s. planes.
Refinement. Refinement of *F*^2^ against ALL reflections. The weighted *R*-factor *wR* and goodness of fit *S* are based on *F*^2^, conventional *R*-factors *R* are based on *F*, with *F* set to zero for negative *F*^2^. The threshold expression of *F*^2^ > σ(*F*^2^) is used only for calculating *R*-factors(gt) *etc*. and is not relevant to the choice of reflections for refinement. *R*-factors based on *F*^2^ are statistically about twice as large as those based on *F*, and *R*- factors based on ALL data will be even larger.

### Fractional atomic coordinates and isotropic or equivalent isotropic displacement parameters (Å^2^)

**Table:**

	*x*	*y*	*z*	*U*_iso_*/*U*_eq_	
S	0.34151 (8)	0.08993 (12)	0.57346 (7)	0.0494 (3)	
N1	0.3802 (2)	0.1397 (4)	0.3670 (2)	0.0477 (7)	
N2	0.4336 (3)	0.2834 (4)	0.4424 (2)	0.0504 (8)	
N3	0.4638 (3)	0.3988 (5)	0.6437 (3)	0.0662 (10)	
H3A	0.5027	0.4894	0.6309	0.079*	
H3B	0.4527	0.3872	0.7149	0.079*	
C1	0.0739 (4)	−0.6083 (7)	0.1831 (5)	0.0932 (17)	
H1B	0.0401	−0.5868	0.0945	0.140*	
H1C	0.0143	−0.6328	0.2186	0.140*	
H1D	0.1250	−0.7105	0.1966	0.140*	
C2	0.1409 (3)	−0.4424 (6)	0.2453 (4)	0.0623 (11)	
C3	0.1475 (3)	−0.2912 (6)	0.1764 (4)	0.0673 (11)	
H3C	0.1101	−0.2917	0.0900	0.081*	
C4	0.2085 (3)	−0.1380 (6)	0.2323 (3)	0.0583 (10)	
H4A	0.2112	−0.0376	0.1834	0.070*	
C5	0.2653 (3)	−0.1341 (5)	0.3606 (3)	0.0449 (8)	
C6	0.2590 (3)	−0.2873 (5)	0.4300 (4)	0.0568 (9)	
H6A	0.2967	−0.2886	0.5164	0.068*	
C7	0.1975 (4)	−0.4375 (5)	0.3720 (4)	0.0640 (11)	
H7A	0.1943	−0.5384	0.4203	0.077*	
C8	0.3296 (3)	0.0279 (4)	0.4202 (3)	0.0414 (7)	
C9	0.4208 (3)	0.2763 (5)	0.5533 (3)	0.0451 (8)	

### Atomic displacement parameters (Å^2^)

**Table:**

	*U*^11^	*U*^22^	*U*^33^	*U*^12^	*U*^13^	*U*^23^
S	0.0640 (6)	0.0540 (6)	0.0378 (5)	−0.0091 (4)	0.0267 (4)	0.0006 (4)
N1	0.0611 (17)	0.0532 (17)	0.0353 (14)	−0.0070 (14)	0.0249 (13)	−0.0057 (12)
N2	0.0683 (18)	0.0538 (18)	0.0387 (15)	−0.0117 (15)	0.0305 (14)	−0.0064 (13)
N3	0.097 (3)	0.070 (2)	0.0441 (17)	−0.0336 (19)	0.0401 (17)	−0.0159 (15)
C1	0.095 (3)	0.086 (4)	0.108 (4)	−0.041 (3)	0.046 (3)	−0.035 (3)
C2	0.063 (2)	0.058 (2)	0.076 (3)	−0.0128 (19)	0.037 (2)	−0.019 (2)
C3	0.072 (3)	0.080 (3)	0.053 (2)	−0.016 (2)	0.024 (2)	−0.015 (2)
C4	0.072 (2)	0.059 (2)	0.046 (2)	−0.011 (2)	0.0240 (18)	−0.0032 (17)
C5	0.0524 (19)	0.0435 (18)	0.0451 (18)	0.0001 (15)	0.0247 (16)	−0.0016 (14)
C6	0.064 (2)	0.051 (2)	0.055 (2)	−0.0024 (18)	0.0193 (18)	0.0026 (18)
C7	0.070 (2)	0.045 (2)	0.084 (3)	−0.0041 (19)	0.034 (2)	0.005 (2)
C8	0.0544 (19)	0.0384 (17)	0.0370 (16)	0.0016 (15)	0.0224 (15)	−0.0011 (13)
C9	0.0540 (19)	0.0482 (19)	0.0380 (17)	−0.0039 (16)	0.0218 (15)	0.0008 (14)

### Geometric parameters (Å, °)

**Table:**

S—C9	1.742 (3)	C2—C7	1.368 (6)
S—C8	1.745 (3)	C2—C3	1.376 (6)
N1—C8	1.292 (4)	C3—C4	1.387 (6)
N1—N2	1.382 (4)	C3—H3C	0.9300
N2—C9	1.309 (4)	C4—C5	1.385 (5)
N3—C9	1.335 (4)	C4—H4A	0.9300
N3—H3A	0.8600	C5—C6	1.390 (5)
N3—H3B	0.8600	C5—C8	1.468 (5)
C1—C2	1.512 (6)	C6—C7	1.380 (5)
C1—H1B	0.9600	C6—H6A	0.9300
C1—H1C	0.9600	C7—H7A	0.9300
C1—H1D	0.9600		
			
C9—S—C8	86.96 (15)	C5—C4—C3	120.3 (4)
C8—N1—N2	114.0 (3)	C5—C4—H4A	119.9
C9—N2—N1	112.0 (3)	C3—C4—H4A	119.9
C9—N3—H3A	120.0	C4—C5—C6	117.9 (3)
C9—N3—H3B	120.0	C4—C5—C8	120.4 (3)
H3A—N3—H3B	120.0	C6—C5—C8	121.6 (3)
C2—C1—H1B	109.5	C7—C6—C5	120.6 (4)
C2—C1—H1C	109.5	C7—C6—H6A	119.7
H1B—C1—H1C	109.5	C5—C6—H6A	119.7
C2—C1—H1D	109.5	C2—C7—C6	121.9 (4)
H1B—C1—H1D	109.5	C2—C7—H7A	119.1
H1C—C1—H1D	109.5	C6—C7—H7A	119.1
C7—C2—C3	117.6 (4)	N1—C8—C5	125.2 (3)
C7—C2—C1	121.2 (4)	N1—C8—S	113.2 (2)
C3—C2—C1	121.2 (4)	C5—C8—S	121.6 (2)
C2—C3—C4	121.8 (4)	N2—C9—N3	124.1 (3)
C2—C3—H3C	119.1	N2—C9—S	113.8 (3)
C4—C3—H3C	119.1	N3—C9—S	122.2 (2)
			
C8—N1—N2—C9	−0.4 (4)	N2—N1—C8—S	0.5 (4)
C7—C2—C3—C4	−0.4 (6)	C4—C5—C8—N1	−30.4 (5)
C1—C2—C3—C4	179.9 (4)	C6—C5—C8—N1	149.7 (4)
C2—C3—C4—C5	0.3 (6)	C4—C5—C8—S	148.1 (3)
C3—C4—C5—C6	0.1 (6)	C6—C5—C8—S	−31.8 (4)
C3—C4—C5—C8	−179.8 (3)	C9—S—C8—N1	−0.3 (3)
C4—C5—C6—C7	−0.3 (5)	C9—S—C8—C5	−179.0 (3)
C8—C5—C6—C7	179.6 (3)	N1—N2—C9—N3	−179.9 (3)
C3—C2—C7—C6	0.2 (6)	N1—N2—C9—S	0.1 (4)
C1—C2—C7—C6	179.9 (4)	C8—S—C9—N2	0.1 (3)
C5—C6—C7—C2	0.2 (6)	C8—S—C9—N3	−179.9 (3)
N2—N1—C8—C5	179.1 (3)		

### Hydrogen-bond geometry (Å, °)

**Table:**

*D*—H···*A*	*D*—H	H···*A*	*D*···*A*	*D*—H···*A*
N3—H3A···N2^i^	0.86	2.13	2.970 (5)	166
N3—H3B···N1^ii^	0.86	2.18	3.025 (4)	166

Symmetry codes: (i) −*x*+1, −*y*+1, −*z*+1; (ii) *x*, −*y*+1/2, *z*+1/2.

## References

[bb1] Allen, F. H., Kennard, O., Watson, D. G., Brammer, L., Orpen, A. G. & Taylor, R. (1987). *J. Chem. Soc. Perkin Trans. 2*, pp. S1–19.

[bb2] Enraf–Nonius (1989). *CAD-4 Software* Enraf–Nonius, Delft, The Netherlands.

[bb3] Han, F., Wan, R., Wu, W.-Y., Zhang, J.-J. & Wang, J.-T. (2007). *Acta Cryst.* E**63**, o717–o718.

[bb4] Harms, K. & Wocadlo, S. (1995). *XCAD4* University of Marburg, Germany.

[bb5] Nakagawa, Y., Nishimura, K., Izumi, K., Kinoshita, K., Kimura, T. & Kurihara, N. (1996). *J. Pestic. Sci* **21**, 195-201.

[bb6] North, A. C. T., Phillips, D. C. & Mathews, F. S. (1968). *Acta Cryst.* A**24**, 351–359.

[bb7] Sheldrick, G. M. (2008). *Acta Cryst.* A**64**, 112–122. 10.1107/S0108767307043930 18156677

[bb8] Wang, Y. G., Cao, L., Yan, J., Ye, W. F., Zhou, Q. C. & Lu, B. X. (1999). *Chem. J. Chin. Univ.* **20**, 1903–1905.

